# Inflammatory serum markers up to 5 years after comprehensive periodontal therapy of aggressive and chronic periodontitis

**DOI:** 10.1007/s00784-018-2398-x

**Published:** 2018-02-27

**Authors:** Tatjana Ramich, Anne Asendorf, Katrin Nickles, Gerhard M. Oremek, Ralf Schubert, Luigi Nibali, Martin Wohlfeil, Peter Eickholz

**Affiliations:** 1 Department of Periodontology, Center for Dentistry and Oral Medicine (Carolinum), Johann Wolfgang Goethe-University Frankfurt/Main, Theodor-Stern-Kai 7, 60596 Frankfurt am Main, Germany; 2 Department of Laboratory Medicine, Centre for Internal Medicine, Hospital of the Johann Wolfgang Goethe-University Frankfurt/Main, Theodor-Stern-Kai 7, 60590 Frankfurt, Germany; 3 Department for Children and Adolescents, Division of Allergology, Pulmonology, and Cystic Fibrosis, Hospital of the Johann Wolfgang Goethe-University Frankfurt/Main, Theodor-Stern-Kai 7, 60590 Frankfurt, Germany; 4 Centre for Oral Clinical Research, Institute of Dentistry, Queen Mary University of London, Turner Street, London, E1 2AD UK

**Keywords:** Leukocyte elastase, C-reactive protein, Lipopolysaccharide-binding protein, Interleukin-6, Aggressive periodontitis, Chronic periodontitis

## Abstract

**Aim:**

The aim of the study is to assess the long-term effect of active periodontal therapy on serum inflammatory parameters in patients with aggressive (AgP) and chronic (ChP) periodontitis in a non-randomised clinical study.

**Methods:**

Twenty-five ChP and 17 AgP were examined clinically prior to (baseline), 12 weeks and 60 months after subgingival debridement of all pockets within 2 days. Systemic antibiotics were prescribed if *Aggregatibacter actinomycetemcomitans* was detected (10 AgP, 8 ChP), flap surgery was rendered if required. Neutrophil elastase (NE), C-reactive protein (CRP), lipopolysaccharide binding protein, interleukin 6, 8, and leukocyte counts were assessed at baseline, 12 weeks and 60 months.

**Results:**

Clinical parameters improved significantly in both groups from 12 weeks to 60 months. Eleven AgP and 18 ChP patients received surgical treatment after the 12 weeks examination. Only 3 patients in each group attended ≥ 2 supportive maintenance visits per year. NE and CRP were significantly higher in AgP than ChP at baseline and 60 months (*p* < 0.01). For leukocyte counts in ChP, significant changes were observed (baseline: 6.11 ± 1.44 nl^−1^; 12 weeks: 5.34 ± 1.40 nl^−1^; 60 months: 7.73 ± 2.89 nl^−1^; *p* < 0.05). Multiple regression analysis identified African origin, surgical treatment and female sex to correlate with better clinical improvement.

**Conclusion:**

Despite comprehensive periodontal treatment, AgP patients exhibit higher NE and CRP levels than ChP patients up to 5 years after therapy.

**Clinical relevance:**

Systemic inflammatory burden in AgP patients is higher than in ChP patients even 5 years after periodontal treatment.

**Electronic supplementary material:**

The online version of this article (10.1007/s00784-018-2398-x) contains supplementary material, which is available to authorized users.

## Introduction

Even everyday practices such as tooth brushing, flossing, and chewing result in frequent bacteraemia in individuals suffering from untreated periodontal disease [1]. Frequent bacteraemia and systemic spread of proinflammatory cytokines [2] from periodontal pockets cause the release of neutrophil elastase (NE) and acute phase proteins (e.g. C-reactive protein: CRP). Thus, serum CRP [3] and NE [4, 5] are elevated in patients with untreated periodontitis compared to healthy controls. Increased serum NE and CRP caused by periodontitis may link periodontal and systemic diseases [cardiovascular disease (CVD), ischemic stroke [6–8], as well as chronic obstructive pulmonary diseases (COPD) [9, 10]].

A recent study compared NE and CRP levels in patients suffering from a similar severity of untreated aggressive and chronic periodontitis. NE and CRP levels in aggressive periodontitis were found to be elevated compared to chronic periodontitis [4]. Nonsurgical anti-infective therapy resulted in significant clinical improvement in both groups [11].

Thorough nonsurgical subgingival debridement results in serum NE reduction in aggressive but not in chronic periodontitis 12 weeks after therapy [12]. However, how long does this effect last? The aim of this study therefore was to evaluate the effect of comprehensive periodontal therapy after up to 5 years on serum inflammatory parameters in patients with aggressive (AgP) and chronic (ChP) periodontitis that had been already reported 12 weeks after nonsurgical therapy [12].

## Material and methods

Originally 60 patients with untreated severe periodontal disease (31 generalised severe ChP; 29 AgP) were recruited at the Department of Periodontology of the Center for Dentistry and Oral Medicine (Carolinum), Johann Wolfgang Goethe-University Frankfurt/Main for anti-infective treatment [11, 12]. After completion of nonsurgical therapy, all patients were offered to participate in supportive periodontal treatment (SPT). Some required additional surgical treatment (residual pocket probing depth [PPD] > 5.5 mm). Five years after completion of nonsurgical treatment all of these patients were invited for re-examination.

- Inclusion criteria (at baseline):
  - ≥ 16 years of age
  - ≥ 20 remaining teeth
  - Written informed consent
- Aggressive periodontitis
  - Patient is clinically healthy, i.e. he or she does not suffer from systemic diseases predisposing to periodontitis (e.g. diabetes mellitus)
  - Pocket probing depths (PPD) ≥ 3.6 mm at more than 30% of sites [According to the Periodontal Screening and Recording (PSR) index [13] and the guidelines for treatment of statutory insured patients in Germany [14] a PPD of 3.5 mm is the threshold for periodontal disease and thus requirement of therapy. The Florida Probe allows measurements to the nearest 0.2 mm. Thus, PPD ≥ 3.6 mm were used as threshold for periodontal disease].
  - Radiographic bone loss ≥ 50% at a minimum of 2 separate teeth
  - Age at time of diagnosis ≤ 35 years
  - Age at time of recruitment ≤ 37 years of age
- Generalised severe chronic periodontitis
  - PPD ≥ 3.6 mm and vertical clinical attachment loss (CAL-V) ≥ 5 mm at more than 30% of sites
  - PPD ≥ 7 mm at a minimum of 4 sites
  - > 35 years of age
- Exclusion criteria:
  - Requirement of systemic antibiotics for any procedure that may cause transitory bacteraemia (e.g. pocket probing)
  - Self-reported chronic disease influencing the serum CRP level (e.g. rheumatoid arthritis, Crohn’s disease or ulcerative colitis)
  - Self-reported infectious disease within the last 8 weeks before examination (history of fever)
  - Any clinically assessed chronic dermal or mucosal inflammatory condition (e.g. lichen planus)
  - Nonsurgical or surgical periodontal treatment within the last 24 months before examination
  - Systemic or topical subgingival antibiotics within the last 8 weeks before examination

At baseline and 5 years after nonsurgical therapy, all patients were asked about their medical history, actual body weight and height as well as about current and past cigarette smoking habits. Patients who reported smoking or had quit smoking for less than 5 years were classified as smokers [15]. Additionally, ethnic origin was recorded [4]. The study complied with the rules of the Declaration of Helsinki and was approved by the Institutional Review Board for Human Studies of the Medical Faculty of the Goethe-University Frankfurt/Main (Application# 188/06). For the 5 years re-examination, a respective amendment was submitted and approved. All participating individuals were informed on risks and benefits as well as the procedures of the re-examination and gave written informed consent.

### Clinical examination

Clinical examinations are reported in detail elsewhere [4, 12]. Gingival Bleeding Index (GBI)[16] and Plaque Control Record (PCR) [17] were assessed at 6 sites per tooth (mesiobuccal, buccal, distobuccal, mesiooral, oral, distooral) at baseline, 12 weeks and 60 months after subgingival debridement (SD). Probing parameters were scored immediately prior to the first session of SD, 12 weeks [12] and 60 months after nonsurgical therapy. PPD (standard probe) and relative vertical probing attachment level (RAL-V) (disk probe) were measured to the nearest 0.2 mm using an electronic probe (Florida Probe, Version 3.2, Gainesville, USA). RAL-V is measured from the base of the pocket to a disk that is located at the incisal margin or the occlusal surface of the respective tooth. Bleeding on probing (BOP) was assessed 30 s after probing. Recession was measured to the nearest 0.5 mm using a manual periodontal probe (PCPUNC 15, HuFriedy, Chicago, USA) from the cemento-enamel junction (CEJ) to the gingival margin. CAL-V was calculated as a sum of PPD and recession. If the CEJ was located apical to the gingival margin, CAL-V was calculated as the PPD minus the distance from the gingival margin to the CEJ. Further, the periodontally inflamed surface area (PISA) was calculated per individual to describe the size of the interface between periodontal pocket and vascular system [18, 19]. For each patient PPD were entered into an Excel sheet that can be downloaded freely (http://www.parsprototo.info/pisa.html).

### Microbiological examination

At baseline as well as 12 weeks [12] and 60 months, subgingival plaque was sampled from the deepest pocket in each of the 4 quadrants. The test site was dried by air and kept dry using cotton rolls. Sterile paper points were inserted to the respective pocket. After 20 s, the paper points were removed. Each patient’s 4 paper points were pooled into one transportation vial. For analysis, a commercially available 16S rRNA gene probe test kit was used (IAI Pado-Test 4.5, Institut für angewandte Immunologie [Institute for Applied Immunology], Zuchwil, Switzerland) aiming at *Aggregatibacter actinomycetemcomitans*, *Porphyromonas gingivalis*, *Tannerella forsythia*, and *Treponema denticola*. This is an oligonucleotide probe test complementary to conservative regions of the 16S rRNA gene which encodes the rRNA that forms the small subunit of the bacterial ribosome. The test was quantitative and its detection limit is 10^3,3^ for *A. actinomycetemcomitans* and 10^4^ for *P. gingivalis*, *T. forsythia*, and *T. denticola*, respectively.

### Blood samples

Twenty ml of blood was sampled from an arm vein at the following times: immediately prior to baseline scoring of probing parameters, 12 weeks [12], and 60 months. Patients were instructed not to be physically active before the blood sample. Intake of food was not standardised. Thereafter, serum levels of high sensitivity CRP, elastase, IL-6, IL-8, LBP concentrations and leukocyte counts were analysed. The assays used for analysis are reported in detail elsewhere [4]. For serum IL-6 at 60 months another test kit (Human IL-6 Flex Set, CBA, Becton Dickinson, CA, USA) was used than for baseline and 12 weeks. For 6 patients IL-6 for baseline or 12 weeks could be analysed with the CBA to calculate a factor of 0.28, 0.23/0.32 (Median, lower/upper quartile) to adjust the T2 values for IL-6.

Blood cells were sent to the Eastman Dental Institute, Division of Microbial Diseases, Periodontology Unit. There DNA was extracted to be analysed for IL-6 single nucleotide polymorphisms (SNP) (rs1800795, C_1839697_20, −174 C/G; rs2069827, C_15860047_10, −1363 G/T)). Homozygous subjects for −174 G and −1363 G allele were defined positive for a supposedly hyper-inflammatory haplotype [20].

### Cell samples

Further a sample of cells from the cheek mucosa was obtained using a foam swab wiped over it for 20 s. The sample was then sent for laboratory analysis to detect the presence of the interleukin-1 composite genotype (GenoType® IL-1, Hain Lifescience GmbH, Nehren, Germany). Positivity for this composite genotype was defined as the presence of at least one copy of ‘allele 2’ for IL1B rs 1143634 (previously reported as IL1B +3953 or +3954) and IL1 A rs 1800587 (previously reported as IL1A-889) [21].

### Anti-infective therapy

Anti-infective therapy has been described in detail before [12]. All patients received oral hygiene instructions and professional prophylaxes until the PCR was ≤ 50%. SD was performed in 2 visits on 2 consecutive days. On the first day, the right side (1st and 4th quadrant) was treated, on the following day the left side (2nd and 3rd quadrant). Immediately after local anaesthesia (UDS, Sanofi-Aventis Deutschland GmbH, Frankfurt/Main, Germany) each patient brushed the back of the tongue for 60 s with 1% chlorhexidine (CHX) gel (Chlorhexamed 1% Gel, GlaxoSmithKline, München, Germany) and rinsed 2 times for 60 s with 10 ml of 0.12% CHX solution (ParoEx, John O. Butler, Kriftel, Germany). For the last 10 s patients were advised to gargle. All teeth exhibiting PPD ≥ 3.6 mm were subgingivally debrided using sonic scalers (Sonicsys, KaVo, Biberach, Germany) and hand instruments. Immediately after instrumentation 1% CHX gel was applied into all debrided pockets 3 times within 10 min [22]. If *A. actinomycetemcomitans* had been detected from subgingival plaque, 500 mg amoxicillin and 400 mg metronidazole were prescribed 3 times daily for 7 days. In case of sensitivity to penicillin, 250 mg ciprofloxacin and 500 mg metronidazole were prescribed 2 times daily for 7 days [23–26]. Six days after accomplishment of SD subgingival application of 1% CHX gel was repeated. For all patients oral home care for 14 days after start of SD included the following: rinsing 2 times daily for 60 s with 10 ml 0.12% CHX solution (ParoEx), then brushing of teeth and the back of the tongue with 1% CHX gel. Six and 12 weeks after subgingival debridement, all patients received oral hygiene instructions and professional prophylaxis.

### Supportive periodontal therapy

SPT was offered to all patients after the 12 weeks re-examination [11, 12]. SPT encompassed the following elements for all patients at each appointment [27]: Assessment of GBI and PCR, re-instruction and re-motivation to effective individual plaque control, professional mechanical plaque removal (PMPR) with hand instruments and polishing of all teeth using rubber cups and polishing paste, application of a fluoride gel. Twice a year a dental status and PPD as well as once a year CAL-V were obtained at 6 sites per tooth. Thirty seconds after probing BOP was recorded. Sites exhibiting PPD = 4 mm and BOP as well as sites with PPD ≥ 5 mm were re-instrumented subgingivally. If a patient exhibited more than 5 to 6 sites that ought to be debrided subgingivally recurrent anti-infective therapy was recommended. Assignment of SPT intervals was performed according to the periodontal risk assessment (PRA) [27–29].

### Statistical analysis

Serum NE and CRP 60 months after therapy were defined as the main outcome variables. Secondary outcome variables were leukocyte counts, LBP, IL-6, and IL-8 as well as PPD reduction, PISA reduction, and CAL-V gain. Analysis was performed per protocol. Missing data were handled using the last observation carried forward method.

For all individuals, the body mass index (BMI) and cigarette pack years were calculated at baseline and 60 months after therapy. Group frequencies (ChP, AgP) were expressed for sex, current smoking, *A. actinomycetemcomitans*-positive, IL-1, IL-6-haplotype-positive, and CRP concentrations (0.1 to 0.3 mg/dl, > 0.3 mg/dl) [30]. Group means and standard deviations were calculated for all normally distributed variables (age, number of remaining teeth, BMI). Further, group medians and lower/upper quartiles were calculated for (pack years, GBI, PCR and BOP at baseline, 12 weeks, and 60 months as well as for the changes between baseline, 12 weeks and 60 months). For all site-based periodontal parameters (PPD, CAL-V, RAL-V) means per individual were calculated at baseline, 12 weeks, and 60 months as well as for changes between baseline, 12 weeks, and 60 months from which group means and standard deviations were calculated. The percentage of sites with PPD 5 mm with BOP or PPD ≥ 6 mm per patient was calculated for baseline, 12 weeks, and 60 months. All bacterial counts were log-transformed and group medians and lower/upper quartiles were calculated for baseline, 12 weeks, and 60 months. Group medians and lower/upper quartiles were calculated for all serum variables at baseline, 12 weeks, and 60 months as well as for changes from baseline to 12 weeks and 60 months as well as from 12 weeks to 60 months. In addition, the frequency of CRP reduction ≥ 0.3 mg/dl from baseline to 12 weeks, and 60 months was calculated. Further comparisons between groups for dichotomous parameters were made by *χ*^2^ or Fisher’s exact test and for all other parameters by Mann-Whitney *U* test. Comparisons within groups for dichotomous parameters were made by *χ*^2^ and for all other parameters by Wilcoxon test or Friedman and in case of significant differences Wilcoxon test.

Using stepwise linear backward multiple regression analysis, factors should be identified that influenced the serum NE, CRP, and LBP at T2 as well as change of PISA from 12 weeks to 60 months. The following independent variables were entered into the models: diagnosis, sex, age, ethnic origin (European, Asian, African), BMI at 60 months, smoking status (current/never and former) at 60 months, self-report of infections within the last 8 weeks prior to the 60 months examination, number of teeth removed due to periodontal reasons and number of teeth treated surgically after 12 weeks, SPT at least once a year, log-transformed numbers of *A. actinomycetemcomitans, P. gingivalis, T. forsythia, T. denticola* at 60 months, PPD at 60 months and PPD reduction from 12 weeks to 60 months. The following parameters were described by dummy variables: diagnosis (ChP = 0, AgP = 1), sex (male = 0, female = 1), ethnicity (European/Asian/African = 0/1/0, European/Asian/African = 0/0/1), smoking status (never and former smoker = 0, current smoker = 1), SPT at least once a year (no = 0, yes = 1). All factors with *p* < 0.05 were kept in the models. For statistical analysis, a PC program (Systat™ for Windows Version 13, Systat Inc., Evanston, USA) was used.

## Results

Twenty-five chronic periodontitis and 17 aggressive periodontitis patients were re-examined at 60 months after SD between September 2012 and November 2014. Six (19.4%) ChP and 12 (41.4%) AgP did not follow the invitation for the 5 years examination (*p* = 0.063). One patient had passed away (ChP), 2 had left the study immediately after 6 weeks (1 AgP), 3 had moved away from Frankfurt or were not available due to their work (2 AgP). Additional 12 had been contacted by phone and/or mail of which 10 did not reply and 2 explicitly refused to participate (9 AgP). Patient characteristics are given in Table 1. One AgP patient had developed a type 2 diabetes mellitus from baseline to 60 months. Therapy rendered additionally to SD is given in Table 2. During SD 1 tooth was removed in one ChP patient. Between 12 weeks and 60 months 25 teeth in 16 ChP and 16 teeth in 6 AgP patients were extracted. After 12 weeks in ChP significantly more teeth were treated surgically than in AgP (Table 2). Six patients reported infections (e.g. tick bite, cold) or treatments within the last 8 weeks prior to the 60 months examination (1 ChP, 5 AgP). However, none reported fever within the last 8 weeks prior to the 60 months examination which had been an exclusion criterion at baseline.

**Table 1:** Patients’ characteristics

Parameters	Chronic periodontitis; ChP (*n* = 25)	Aggressive periodontitis; AgP (*n* = 17)	*p*
Female sex: [*n*]/frequency (%)	10 (40%)	8 (47%)	0.650
Age at re-examination (T2) [years]: mean ± SD	58.2 ± 7.2	35.2 ± 6.6	< 0.001
Ethnicity: [*n*]/frequency (%)			0.185
African	0	3 (18%)	0.059
Asian	2 (8%)	2 (12%)	1.000
European	23 (92%)	12 (70%)	0.202
Remaining teeth [*n*]: mean ± SD			
Baseline	26.2 ± 2.9	29.2 ± 2.5	< 0.001
5 years after nonsurgical therapy	25.2 ± 2.7	28.3 ± 2.5	< 0.001
Interleukin 1 haplotype [*n*]/frequency (%)	5 (20%)	5 (29%)	0.714
Interleukin 6 haplotype [*n*]/frequency (%)	11 (44%)	10 (58%)	0.346
Current smokers [*n*]/frequency (%)			
Baseline	7 (28%)	4 (24%)	0.746
5 years after nonsurgical therapy	5 (20%)	2 (12%)	0.482
Pack years: median (lower/upper quartile)	1.4 (0/19.3)	0 (0/1.2)	0.060
Body mass index [kg/m^2^]: mean ± SD			
Baseline	25.7 ± 4.0	25.8 ± 3.8	0.929
5 years after nonsurgical therapy	25.9 ± 4.8	27.5 ± 5.8	0.343

**Table 2:** Therapy additional to nonsurgical anti-infective treatment (subgingival debridement: SD; supportive periodontal treatment: SPT)

Parameters	Chronic periodontitis; ChP (*n* = 25)	Aggressive periodontitis; AgP (*n* = 17)	*p*
Extractions between baseline and 3 months: median (lower/upper quartile)	0 (0/1.3)	0 (0/0)	0.410
Extractions between 3 months and 5 years: median (lower/upper quartile)	1.0 (0/1.3)	0 (0/2.3)	0.442
Systemic antibiotic treatment adjunctive to SD Patients: [*n*]/frequency (%)	8 (32%)	10 (59%)	0.085
Surgical therapy between 3 months and 5 years			
Patients: [*n*]/frequency (%)	18 (72%)	11 (65%)	0.616
Teeth per patient: median (lower/upper quartile)	9.0 (1.5/13.0)	5.0 (0/7.3)	0.022
SPT			
Total number of visits: median (lower/upper quartile)	10.0 (7.0/13.0)	9.0 (4.3/10.5)	0.142
≥ 3 visits per year: [*n*]/frequency (%)	1 (4%)	1 (6%)	1.000
2 visits per year: [*n*]/frequency (%)	2 (8%)	2 (12%)	1.000
1 visit per year: [*n*]/frequency (%)	8 (32%)	4 (24%)	0.551

Clinical variables (BOP, PPD, % of sites with PPD 5 mm with BOP or PPD ≥ 6 mm, RAL-V, PISA) were significantly improved in both groups from baseline to 12 weeks and from 12 weeks to 60 months (Table 3). At baseline mean PPD, % of sites with PPD 5 mm with BOP or PPD ≥ 6 mm and mean PISA are significantly higher in ChP than in AgP (Table 3). Twelve weeks and 60 months after SD, these differences have disappeared (Table 3). Serum NE levels were significantly higher in AgP than in ChP at baseline and at 60 months. A significant difference was observed regarding change of serum NE at 12 weeks between AgP (− 2.1 ng/ml) and ChP (0.0 ng/ml) (*p* = 0.03) (Table 4). Median serum CRP levels were higher in AgP than in ChP at all re-examinations (Table 4). LPS is significantly higher in AgP than ChP and exhibits better reduction in AgP than ChP (Table 5). IL-6 does change in neither group during the observation period. In ChP IL-8 is significantly increased at 12 weeks compared to AgP and significantly drops down to 60 months. In AgP changes of IL-8 are small and insignificant (Table 6). Backward stepwise linear multiple regression analysis identified AgP, African origin, and age to be positively associated with serum NE 60 months after SD (Table 7). Serum CRP 60 months after SD is positively correlated to AgP (Table 7). LBP at 60 months is negatively associated with Asian origin and at least 1 SPT visit per year, whereas AgP, age, and BMI at 60 months are positively related (Table 7).

**Table 3:** Individuals’ periodontal variables and change of periodontal variables after therapy (CAL-V: clinical vertical attachment level; RAL-V: relative vertical attachment level; PISA: periodontally inflamed surface area)

Parameters		Chronic periodontitis; ChP (*n* = 25)	Aggressive periodontitis; AgP (*n* = 17)	*p*
Gingival Bleeding Index [%]	Baseline	15.0 (5.8/20.0)	10.0 (6.8/18.0)	0.572
median (lower/upper quartile)	12 weeks	4.0 (2.8/9.0)^a^	5.0 (2.0/6.3)^b^	0.857
	60 months	3.0 (0/7.0)^a^	5.0 (1.8/10.5)	0.238
Plaque Control Record [%]	Baseline	30.0 (24.5/38.0)	40.0 (27.3/45.3)	0.119
median (lower/upper quartile)	12 weeks	23.0 (18.0/42.0)	27.0 (14.8/30.5)	0.442
	60 months	40.0 (20.0/50.0)	25.0 (16.8/39.0)	0.243
Bleeding on probing [%]	Baseline	52.0 (43.8/60.8)	43.0 (38.3/46.8)	0.027
median (lower/upper quartile)	12 weeks	27.0 (17.8/33.0)^a^	22.0 (18.8/26.5)^a^	0.434
	60 months	11.0 (5.0/19.0)^a/c^	7.0 (5.8/11.5)^a/c^	0.258
Probing depth (PPD) [mm]	Baseline	3.9 ± 0.6	3.4 ± 0.6	0.018
mean ± SD	12 weeks	2.6 ± 0.4^a^	2.5 ± 0.3^a^	0.455
PPD reduction [mm]	12 weeks	− 1.3 ± 0.4	− 0.9 ± 0.5	0.009
mean ± SD	60 months	2.0 ± 0.5^a/c^	1.8 ± 0.4^a/c^	0.258
PPD reduction [mm]	60 months	− 1.9 ± 0.6	− 1.6 ± 0.6	0.089
Sites with PPD 5 mm and BOP or PPD ≥ 6 mm mm [%]	Baseline	32.1 (24.9/36.5)	21.0 (13.4/25.5)	0.003
median (lower/upper quartile)	12 weeks	6.7 (4.5/10.4)^a^	4.7 (2.2/9.6)^a^	0.155
	60 months	2.2 (0.7/3.7)^a/d^	1.3 (0/3.9)^a/d^	0.279
Attachment level [mm] (CAL-V)	Baseline	4.1 ± 0.9	2.7 ± 1.2	0.001
(RAL-V)	Baseline	11.3 ± 1.1	10.0 ± 1.4	0.003
mean ± SD	12 weeks	10.8 ± 1.0^a^	9.5 ± 1.2^a^	0.001
Attachment gain [mm] (ΔRAL-V)	12 weeks	0.5 ± 0.3	0.5 ± 0.4	0.984
mean ± SD	60 months	10.4 ± 1.0^a/c^	9.1 ± 1.3^a/d^	0.002
Attachment gain [mm] (ΔRAL-V)	60 months	0.9 ± 0.5	0.9 ± 0.5	0.832
PISA [mm^2^]	Baseline	1483.2 ± 448.9	1156.9 ± 469.9	0.031
mean ± SD	12 weeks	432.5 ± 223.4^a^	396.0 ± 163.1^a^	0.544
	60 months	146.1 ± 120.0^a/c^	109.7 ± 93.6^a/c^	0.277

Significantly different to baseline: ^a^(*p* < 0.001); ^b^(*p* < 0.05)

Significantly different to 12 weeks: ^c^(*p* < 0.001); ^d^(*p* < 0.05)

**Table 4:** Individuals’ serum neutrophil elastase (NE) and C-reactive protein (CRP) concentrations

Parameters		Chronic periodontitis; ChP (*n* = 25)	Aggressive periodontitis; AgP (*n* = 17)	ChP/AgP *p*
Neutrophil elastase [ng/ml]	Baseline	10.9 (7.7/30.3)	36.0 (25.4/37.8)	0.002
median (lower/upper quartile)	12 weeks	18.8 (7.2/36.1)	33.9 (22.0/36.5)	0.075
Change baseline to 12 weeks		0.0 (− 1.4/5.9)	− 2.1 (− 4.6/− 0.6)	0.030
	60 months	14.3 (9.8/22.5)	22.4 (17.3/37.1)	0.006
Change baseline to 60 months		2.1 (− 6.6/11.1)	− 7.0 (− 20.8/10.2)	0.473
Change 12 weeks to 60 months		− 1.0 (− 21.1/7.9)	2.4 (− 17.9/9.8)	0.818
CRP [mg/dl]	Baseline	0.10 (0.08/0.16)	0.24 (0.14/0.48)	0.001
median (lower/upper quartile)	12 weeks	0.11 (0.07/0.22)	0.28 (0.14/0.45)	0.007
Change baseline to 12 weeks		0.01 (− 0.03/0.04)	0.0 (− 0.09/0.09)	0.778
	60 months	0.10 (0.06/0.31)	0.43 (0.25/0.93)	0.005
Change baseline to 60 months		− 0.02 (− 0.07/0.17)	0.13 (− 0.09/0.66)	0.522
Change 12 weeks to 60 months		0.01 (− 0.08/0.14)	0.21 (− 0.08/0.71)	0.324
CRP reduction ≥ 0.3 mg/dl [*n*]/frequency (%) baseline to 12 weeks		0 (0%)	3 (18%)	0.059
Baseline to 60 months [n]/frequency (%)		1 (4%)	3 (18%)	0.286
CRP < 0.1 mg/dl	Baseline	10 (40%)	0 (0%)	0.003
[n]/frequency (%)	12 weeks	11 (44%)	2 (12%)	0.027
	60 months	12 (48%)	2 (12%)	0.020
CRP 0.1 to 0.3 mg/dl [*n*] (%)	Baseline	13 (52%)	10 (59%)	0.663
[*n*]/frequency (%)	12 weeks	10 (40%)	7 (41%)	0.939
	60 months	7 (28%)	3 (18%)	0.490
CRP > 0.3 mg/dl [*n*] (%)	Baseline	2 (8%)	7 (41%)	0.019
[*n*]/frequency (%)	12 weeks	4 (16%)	8 (47%)	0.041
	60 months	6 (24%)	12 (70%)	0.003

**Table 5:** Individuals’ serum leukocyte counts and lipopolysaccharide (LPS) binding protein concentrations; median (lower/upper quartile) (LPS: lipopolysaccharide); mean ± standard deviation

Parameters		Chronic periodontitis; ChP (*n* = 25)	Aggressive periodontitis; AgP (*n* = 17)	ChP/AgP *p*
Leukocyte count [nl^−1^]	Baseline	6.11 ± 1.44	6.46 ± 2.65	0.616
	12 weeks	5.34 ± 1.40^a^	6.11 ± 1.90	0.142
Change baseline to 12 weeks		− 0.77 ± 1.19	− 0.35 ± 1.72	0.398
	60 months	7.73 ± 2.89^b^	8.92 ± 4.73	0.361
Change baseline to 60 months		1.62 ± 3.65	2.46 ± 6.05	0.614
Change 12 weeks to 60 months		2.39 ± 3.50	2.81 ± 5.41	0.777
LPS binding protein [μg/ml]	Baseline	28.46 ± 16.45	39.17 ± 13.94	0.029
	12 weeks	27.76 ± 13.57	31.45 ± 12.61	0.372
Change baseline to 12 weeks		− 0.70 ± 19.19	− 7.71 ± 15.77	0.206
	60 months	31.15 ± 8.68	31.19 ± 11.29	0.990
Change baseline to 60 months		2.69 ± 17.31	− 7.98 ± 14.83	0.039
Change 12 weeks to 60 months		3.39 ± 13.90	− 0.27 ± 17.58	0.479

Significantly different to baseline: ^a^(*p* < 0.05)

Significantly different to 12 weeks: ^b^(*p* < 0.05)

**Table 6:** Individuals’ serum interleukin 6 and 8 concentrations; median (lower/upper quartile)

Parameters		Chronic periodontitis; ChP (*n* = 25)	Aggressive periodontitis; AgP (*n* = 17)	ChP/AgP *p*
Interleukin 6 [pg/ml]	Baseline	1.6 (1.3/2.3)	1.1 (0.9/1.8)	0.074
	12 weeks	1.3 (0.9/2.7)	1.4 (0.9/1.8)	0.787
Change baseline to 12 weeks		− 0.2 (− 0.4/0.3)	0.1 (− 0.1/0.4)	0.976
	60 months	1.6 (1.4/1.8)	1.4 (1.2/1.8)	0.174
Change baseline to 60 months		0 (− 0.6/0.3)	0.3 (− 0.5/0.7)	0.205
Change 12 weeks to 60 months		0.2 (− 1.1/0.6)	0.1 (− 0.1/0.4)	0.768
Interleukin 8 [pg/ml]	Baseline	19.0 (14.3/32.0)	19.0 (11.5/23.3)	0.329
	12 weeks	28.0 (22.0/43.0)	19.0 (10.8/33.3)	0.023
Change baseline to 12 weeks		4.0 (− 2.8/14.5)	0.0 (− 10.0/16.0)	0.457
	60 months	16.4 (12.3/20.8)^c^	16.0 (12.9/19.6)	0.547
Change baseline to 60 months		− 1.6 (− 15.6/3.1)	− 2.0 (− 6.6/4.1)	0.788
Change 12 weeks to 60 months		− 9.4 (− 18.4/− 5.8)	− 1.0 (− 13.4/4.8)	0.079

Significantly different to 12 weeks: ^c^(*p* < 0.001)

**Table 7:** Backward stepwise multiple regression analyses

a) log-transformed serum elastase 60 months after nonsurgical therapy; *n* = 42; *R*^2^ = 0.309; *R*^2^ adjusted = 0.255; standard error of estimate = 0.184			
	*b*	s.e. (*b*)	*p*
Constant	0.669	0.252	0.012
Aggressive periodontitis	0.360	0.117	0.004
African origin	0.288	0.127	0.029
Age	0.010	0.005	0.044
Analysis of variance: *p* = 0.003			
b) log-transformed serum CRP 60 months after nonsurgical therapy; *n* = 42; *R*^2^ = 0.190; *R*^2^ adjusted = 0.170; standard error of estimate = 0.512			
Constant	− 0.899	0.102	< 0.001
Aggressive periodontitis	0.493	0.161	0.004
Analysis of variance: *p* = 0.004			
c) Lipopolisaccharide-binding protein 60 months after nonsurgical therapy; *n* = 42; *R*^2^ = 0.438; *R*^2^ adjusted = 0.360; standard error of estimate = 7.744			
Constant	− 0.872	11.136	0.431
Aggressive periodontitis	11.420	4.963	0.027
Asian origin	− 8.982	4.189	0.039
Age	0.545	0.184	0.005
Body mass index 60 months after therapy	0.553	0.237	0.025
At least 1 supportive periodontal treatment visit per year	− 8.072	2.716	0.005
Analysis of variance: *p* = 0.001			
d) Reduction of PISA from 12 weeks to 60 months; *n* = 42; *R*^2^ = 0.374; *R*^2^ adjusted = 0.324; standard error of estimate = 138.473			
Constant	− 165.198	35.307	< 0.001
African origin	− 187.202	83.849	0.032
Female sex	− 103.235	47.514	0.036
Additional surgical treatment after T1	− 9.566	4.314	0.033
Analysis of variance: *p* < 0.001			

Another backward stepwise linear multiple regression analysis identified African origin, female sex, and number of teeth additionally surgically treated after 12 weeks to be associated with more favourable PISA reduction (Table 7).

At baseline 18 patients (ChP: 8; AgP: 10) were positive for *A. actinomycetemcomitans* and received adjunctive systemic antibiotics. Twelve weeks after SD only 2 patients (both ChP) were still positive for *A. actinomycetemcomitans*. One had already been positive at baseline the other not. At the 60 months examination *A. actinomycetemcomitans* was detected in 6 patients (ChP: 3; AgP: 3). Two AgP had been positive at baseline. All others had been *A. actinomycetemcomitans* negative at baseline and 12 weeks. *A. actinomycetemcomitans* was detected in ChP in significantly lower numbers than in AgP. SD resulted in significant reduction of *A. actinomycetemcomitans* in AgP after 12 weeks and 60 months. Whereas numbers of *P. gingivalis* were significantly reduced in AgP after 12 weeks and 60 months, numbers ChP relapsed from 12 weeks to 60 months. Numbers of *T. forsythia* and *T. denticola* were significantly reduced by therapy in ChP and AgP (Table 8).

**Table 8:** Log-transformed numbers of *A. actinomycetemcomitans, P. gingivalis, T. forsythia, T. denticola*; median (lower/upper quartile)

Parameters		Chronic periodontitis; ChP (*n* = 25)	Aggressive periodontitis; AgP (*n* = 17)	ChP/AgP *p*
*A. actinomycetemcomitans*	Baseline	0 (0/4.78)	4.56 (0/5.59)	0.034
	12 weeks	0 (0/0)	0 (0/0)^b^	0.238
	60 months	0 (0/0)	0 (0/0)^b^	0.570
*P. gingivalis*	Baseline	6.83 (6.40/6.96)	6.48 (5.55/6.93)	0.323
	12 weeks	1.04 (0/6.47)^a^	0.28 (0.14/0.45)^b^	0.180
	60 months	5.72 (0/6.70)^b^	0 (0/4.55)^b^	0.004
*T. forsythia*	Baseline	6.71 (6.49/6.88)	6.73 (6.26/6.89)	0.608
	12 weeks	5.91 (0/6.45)^a^	5.28 (0/5.96)^b^	0.409
	60 months	6.25 (4.57/6.69)^b^	5.85 (0/6.36)^b^	0.239
*T. denticola*	Baseline	6.36 (6.02/6.67)	6.35 (6.19/6.57)	0.798
	12 weeks	5.72 (0/6.13)^b^	5.18 (0/5.38)^b^	0.140
	60 months	5.77 (0/6.29)^b^	4.85 (0/5.98)^b^	0.147

Significantly different to baseline: ^a^(*p* < 0.001); ^b^(*p* < 0.05)

## Discussion

Patients suffering from ChP and AgP were treated by SD of all pockets ≥ 3.6 mm within 2 days. Clinical parameters improved significantly in both groups from baseline to 12 weeks and then further during surgical and maintenance therapy from 12 weeks to 60 months. Eleven AgP and 18 ChP patients received surgical treatment after the 12 weeks examination. Only 3 patients in each group attended ≥ 2 supportive maintenance visits per year. Multiple regression analysis revealed African origin, surgical treatment and female sex to correlate with better clinical improvement. Despite comprehensive therapy, AgP patients exhibited higher NE and CRP levels than ChP patients up to 5 years after periodontal treatment.

Bacteraemia from periodontal pockets and the resulting systemic spread of proinflammatory cytokines cause an acute host response including the production of IL-6 which induces the liver to produce CRP and other acute-phase proteins [12, 31, 32]. In established gingivitis and periodontitis, the parakeratinized and ulcerated pocket epithelium functions as an easy port of entry for oral microorganisms. Combining the pocket walls of all periodontally compromised teeth in an untreated patient, the wound surface, due to periodontitis, is estimated to be as large as 8 to 20 cm^2^ [33]. Different from Eickholz et al. (2013) [12], in the present study PISA was calculated for each patient and examination. At baseline the respective values ranged from 11 to 16 cm^2^ in ChP and from 8 to 13 cm^2^ in AgP. PISA was significantly larger in ChP than in AgP, whereas NE and CRP were significantly elevated in AgP as compared to ChP. The fact that despite similar clinical parameters (PPD and sum of PPD) NE and CRP were significantly elevated in AgP compared to ChP had been shown for the complete cohorts recently [12]. The difference between ChP and AgP can neither be explained by the severity of disease nor the size of the wound (PISA).

SD in this study was effective. It resulted in significant mean PPD reduction (ChP: − 1.3 mm; AgP: − 0.9 mm) and attachment gain (0.5 mm in both groups) from baseline to 12 weeks. These results are in accordance with results reported by other groups [34–36]. Interestingly, from 12 weeks to 60 months (5 years after baseline), there is additional significant improvement of PPD reduction (ChP: − 0.3 mm; AgP: − 0.7 mm) and attachment gain (0.4 mm in both groups). Perhaps 12 weeks after SD are too early to assess the complete treatment effect (Harks et al. 2015). However, 18 ChP and 11 AgP patients received additionally to SD surgical treatment after the 12 weeks examination on average in 9 (ChP) and 5 (AgP) teeth per patient, respectively. Number of teeth additionally surgically treated after the 12 weeks examination was identified to be associated with reduction of PISA from 12 weeks to 60 months. Thus, additional surgical treatment may also explain the additional clinical improvement from 12 weeks to 60 months.

Twelve weeks after SD plus adjunctive antibiotics *A. actinomycetemcomitans* was suppressed below the detection limit in 16 of 18 patients. After 12 weeks and 60 months *A. actinomycetemcomitans* was detected in patients that had been negative at baseline or at 12 weeks. This may be explained by numbers close below the detection limit that may have increased in particular from 12 weeks to 60 months. Log-transformed numbers of *A. actinomycetemcomitans* were higher in AgP than in ChP at baseline which reflects the literature [37] and were reduced significantly most likely due to adjunctive use of antibiotics which was restricted to patients *A. actinomycetemcomitans*-positive at baseline. Treatment was effective to suppress *A. actinomycetemcomitans* below detection limits in AgP. *P. gingivalis* was also significantly reduced in both groups after 12 weeks. However, in ChP *P. gingivalis* numbers rebounded in some patients and resulted in significantly higher numbers 60 months after therapy. This may be due to the fact that in fewer ChP patients adjunctive antibiotics were used than in AgP (32 vs. 59%).

Untreated periodontitis is associated with elevated NE and CRP and thus may contribute to the risk for CVD and COPD. It had been demonstrated that periodontal treatment reduces serum levels of NE in AgP but not in ChP. This may reduce the respective risks. In this study, NE was reduced at 12 weeks to 60 months compared to baseline. However, the differences were not significant. Further, 18% of AgP patients exhibited a CRP reduction ≥ 0.3 mg/dl from 12 weeks to 60 months compared to 4% in the ChP group. However, this difference was also not significant. From 12 weeks to 60 months, 6 ChP and 12 AgP patients were lost to analysis which represents 27% of the whole sample. The particularly large loss in the AgP group has deteriorated test power and may explain that the reduction of NE from baseline to 12 weeks and 60 months fails to be significant. Interestingly the erosion rate in AgP is larger compared to ChP. Ramseier et al. (2014) found age to correlate with better compliance in SPT patients [28]. In our study by definition AgP were younger than ChP patients. This may explain the higher dropout rate in AgP.

LBP is significantly higher in AgP than ChP and exhibits better reduction in AgP than ChP. Thus, LBP reacts similar to NE and CRP. However, the differences are significant despite substantial erosion of patients. At 60 months LBP levels are quite similar in both groups representing effective control of infection mirroring clinical improvement. Even at 60 months LBP is associated with AgP and BMI. At least 1 SPT visit per year is associated with lower serum LBP which indicates better infection control in regular SPT.

Interestingly compliance even in patients attending the 5 years re-examination of this study is low in general. Median total numbers of SPT visits (ChP: 10; AgP: 9) within 5 years could stand for 1 to 2 SPT visits per year. However, only 32% of ChP and 24% of AgP patients attended at least 1 visit per year. In ChP 5 years plaque levels are quite high. This indicates a low degree of adherence to recommended SPT schedules. Perhaps better SPT adherence is required to keep serum levels of inflammatory serum parameters low.

This study clearly has some weaknesses. First of all the original study had just enough participants to fulfil the required minimal sample size to show differences with regard to NE and CRP [12]. Due to a substantial dropout rate from 12 weeks to 60 months, test power was deteriorated. With regard to the limited sample size, another issue is the different treatments that where applied (adjunctive antibiotics, flap surgery as required). However, all patients were treated consequently according to a consistent treatment concept. Further, until now, the number of studies evaluating the effect of periodontal treatment on serum levels of inflammatory parameters is limited [32, 38–41]. Even more limited is the number of reports on serum inflammatory parameters in AgP [4, 25, 42]. The remarkable strength of this study is the re-examination up to 5 years after comprehensive periodontal treatment including inflammatory serum parameters. To the best of our knowledge, this is the first study to report long-term effects on inflammatory serum parameters.

Within the limitations of the present study, the following conclusion may be drawn: Despite comprehensive periodontal therapy and significant clinical improvement NE and CRP levels in patients with aggressive periodontitis are elevated compared to patients with untreated chronic periodontitis. However, further research is required to confirm this observation that may be used to generate the hypothesis that in AgP the systemic inflammatory burden is less easily to influence by periodontal treatment.

## Electronic supplementary material

ESM 1 (DOCX 43 kb)
